# Comparison of immunoassay- with mass spectrometry-derived p-tau quantification for the detection of Alzheimer’s disease pathology

**DOI:** 10.1186/s13024-023-00689-2

**Published:** 2024-01-07

**Authors:** Joseph Therriault, Marcel S. Woo, Gemma Salvadó, Johan Gobom, Thomas K. Karikari, Shorena Janelidze, Stijn Servaes, Nesrine Rahmouni, Cécile Tissot, Nicholas J. Ashton, Andréa Lessa Benedet, Laia Montoliu-Gaya, Arthur C. Macedo, Firoza Z. Lussier, Jenna Stevenson, Paolo Vitali, Manuel A. Friese, Gassan Massarweh, Jean-Paul Soucy, Tharick A. Pascoal, Erik Stomrud, Sebastian Palmqvist, Niklas Mattsson-Carlgren, Serge Gauthier, Henrik Zetterberg, Oskar Hansson, Kaj Blennow, Pedro Rosa-Neto

**Affiliations:** 1 Translational Neuroimaging Laboratory, McGill University Research Centre for Studies in Aging, Alzheimer’s Disease Research Unit, Douglas Research Institute, Le Centre Intégré Universitaire de Santé Et de Services Sociaux (CIUSSS) de l’Ouest-de-L’Île-de-Montréal, 6875 La Salle Blvd - FBC Room 3149, Montréal, Québec H4H 1R3 Canada; 2 Department of Neurology and Neurosurgery, McGill University, Montreal, QC H3A 2B4 Canada; 3 Department of Neurology, Institute of Neuroimmunology and Multiple Sclerosis, University Medical Center Hamburg-Eppendorf, Hamburg, 20251 Germany; 4 Department of Clinical Sciences Malmö, Clinical Memory Research Unit, Lund University, Lund, Sweden; 5 Department of Psychiatry and Neurochemistry, Institute of Neuroscience and Physiology, The Sahlgrenska Academy, University of Gothenburg, Mölndal, S-431 80 Sweden; 6 Clinical Neurochemistry Laboratory, Sahlgrenska University Hospital, Mölndal, S-431 80 Sweden; 7 Department of Psychiatry, University of Pittsburgh School of Medicine, Pittsburgh, 15213 USA; 8 Wallenberg Centre for Molecular Medicine, University of Gothenburg, Gothenburg, S-413 45 Sweden; 9 King’s College London, Institute of Psychiatry, Psychology and Neuroscience, Maurice Wohl Institute Clinical Neuroscience Institute, London, SE5 9RT UK; 10 NIHR Biomedical Research Centre for Mental Health and Biomedical Research Unit for Dementia at South London and Maudsley NHS Foundation, London, SE5 8AF UK; 11 Memory Clinic, Skåne University Hospital, Malmö, Sweden; 12 Wallenberg Center for Molecular Medicine, Lund University, Lund, Sweden; 13 Department of Neurodegenerative Disease, UCL Institute of Neurology, London, WC1N 6BG UK; 14 UK Dementia Research Institute at UCL, London, WC1N 6BG UK; 15 Hong Kong Center for Neurodegenerative Diseases, Clear Water Bay, Hong Kong, China; 16 Wisconsin Alzheimer’s Disease Research Center, University of Wisconsin School of Medicine and Public Health, University of Wisconsin-Madison, Madison, WI 53792 USA

## Abstract

**Background:**

Antibody-based immunoassays have enabled quantification of very low concentrations of phosphorylated tau (p-tau) protein forms in cerebrospinal fluid (CSF), aiding in the diagnosis of AD. Mass spectrometry enables absolute quantification of multiple p-tau variants within a single run. The goal of this study was to compare the performance of mass spectrometry assessments of p-tau_181_, p-tau_217_ and p-tau_231_ with established immunoassay techniques.

**Methods:**

We measured p-tau_181_, p-tau_217_ and p-tau_231_ concentrations in CSF from 173 participants from the TRIAD cohort and 394 participants from the BioFINDER-2 cohort using both mass spectrometry and immunoassay methods. All subjects were clinically evaluated by dementia specialists and had amyloid-PET and tau-PET assessments. Bland–Altman analyses evaluated the agreement between immunoassay and mass spectrometry p-tau_181_, p-tau_217_ and p-tau_231_. P-tau associations with amyloid-PET and tau-PET uptake were also compared. Receiver Operating Characteristic (ROC) analyses compared the performance of mass spectrometry and immunoassays p-tau concentrations to identify amyloid-PET positivity.

**Results:**

Mass spectrometry and immunoassays of p-tau_217_ were highly comparable in terms of diagnostic performance, between-group effect sizes and associations with PET biomarkers. In contrast, p-tau_181_ and p-tau_231_ concentrations measured using antibody-free mass spectrometry had lower performance compared with immunoassays.

**Conclusions:**

Our results suggest that while similar overall, immunoassay-based p-tau biomarkers are slightly superior to antibody-free mass spectrometry-based p-tau biomarkers. Future work is needed to determine whether the potential to evaluate multiple biomarkers within a single run offsets the slightly lower performance of antibody-free mass spectrometry-based p-tau quantification.

**Supplementary Information:**

The online version contains supplementary material available at 10.1186/s13024-023-00689-2.

## Introduction

Alzheimer’s disease (AD) is defined by cerebral amyloid-β plaques and tau neurofibrillary tangles at autopsy, which differentiate AD from other neurodegenerative diseases [1, 2]. In vivo, assessments of phosphorylated tau (p-tau) also distinguish AD from other neurodegenerative conditions [3, 4] and display good correlations with both amyloid-PET and tau-PET [5, 6]. Fluid biomarkers of AD pathology are anticipated to have important roles in the differential diagnosis of AD, for determining eligibility for clinical trials [7] and for disease-modifying therapies [8].

Several measurement techniques exist for the quantification of p-tau in biofluids [9]. Recent progress in antibody-based immunoassay technology has enabled quantification of very low concentrations of p-tau proteins in cerebrospinal fluid (CSF) [6, 10–13]. However, antibody-based measurement is highly dependent on the quality and availability of antibodies. Recent years have also seen an increase in kit costs for many immunoassays. Mass spectrometry, in contrast, theoretically enables absolute quantification of multiple target proteins in an antibody-independent manner, but requires highly expensive mass spectrometry instruments. Additionally, the technology allows for efficient multiplexing, *i.e.*, quantification of multiple different analytes in a single run [14].

The temporal ordering of p-tau abnormality [12, 15–17], as well as their preferential association with amyloid-β plaques and tau neurofibrillary tangles [18, 19], suggests that assessing multiple p-tau species may be useful for tracking disease severity in AD [20, 21]. Similarly, measuring multiple analytes in CSF at the same time increases the ability to identify individuals at high risk for cognitive decline [22]. However, due to the relative novelty of antibody-free mass spectrometry assays for p-tau, comparisons with more established p-tau biomarkers are required. Here, we compared novel mass spectrometry-based quantification of p-tau_181_, p-tau_217_ and p-tau_231_ with established antibody-based measurements of p-tau_181_, p-tau_217_ and p-tau_231_.

## Methods

### Participants

#### TRIAD

We assessed 173 participants from the Translational Biomarkers of Aging and Dementia (TRIAD) [23] cohort: 23 cognitively unimpaired young adults (CUY), 74 cognitively unimpaired (CU) older adults, 36 individuals with Mild Cognitive Impairment (MCI), 24 individuals with Alzheimer’s clinical syndrome (AD) and 16 participants with other neurodegenerative diseases (OND). All participants had CSF assessments of p-tau_181_, p-tau_217_ and p-tau_231_, from both immunoassays and mass spectrometry. All participants were also evaluated with amyloid-PET with [^18^F]AZD4694 and tau-PET with [^18^F]MK6240. Clinical evaluations of participants included a review of their medical history and an interview with the participant and their study partner, a neurological examination by a physician and a neuropsychological examination. Participants were approached consecutively, and data was collected prospectively from October 2017 to August 2021. CU individuals had no objective cognitive impairment and a Clinical Dementia Rating (CDR) score of 0. Cognitively impaired (CI) participants had objective cognitive impairment and a CDR score of 0.5, 1 or 2. Participants were excluded from this study if they had systemic conditions which were not adequately controlled through a stable medication regimen. Other exclusion criteria were active substance abuse, recent head trauma, recent major surgery, or MRI/PET safety contraindications. The study was approved by the Montreal Neurological Institute PET working committee and the Douglas Mental Health University Institute Research Ethics Board. Written informed consent was obtained for all participants. The present study followed the Strengthening the Reporting of Observational Studies in Epidemiology (STROBE) reporting guidelines.

#### BioFINDER-2

We assessed 394 individuals from the prospective BioFINDER-2 study. This group comprised individuals with mild cognitive impairment (MCI), AD with dementia, various other neurodegenerative conditions, and cognitively unimpaired (CU) individuals. Individuals with AD met the diagnostic criteria outlined in the Diagnostic and Statistical Manual of Mental Disorders [Fifth Edition] [24] in addition to having positive amyloid-beta (Aβ) biomarker results [2]. The inclusion criteria for other neurodegenerative diseases encompassed meeting the criteria for frontotemporal dementia, Parkinson’s disease (PD), PD with dementia, subcortical vascular dementia, progressive supranuclear palsy, multiple system atrophy, or semantic variant primary progressive aphasia, as previously described [25]. CU participants had to not meet criteria for MCI or dementia, showing no history of cognitive decline over time and possessing a CDR score of 0. Recruitment occurred at Skåne University Hospital between April 2017 and September 2019. All participants underwent the Mini-Mental State Examination to assess overall cognition. Ethical approval was granted by the Regional Ethical Committee in Lund, Sweden.

### CSF biomarker quantification

Collection of CSF samples has been reported previously for the TRIAD cohort [15] and BioFINDER-2 cohort [10]. In the TRIAD cohort, CSF concentrations of p-tau_181_, p-tau_217_ and p-tau_231_ were quantified using custom Single molecule array (Simoa; Simoa HD-X instruments, Quanterix, Billerica, MA, USA) assays, as previously described [13, 26]. In the BioFINDER-2 cohort, antibody-based CSF quantification of p-tau_181_ and p-tau_217_ were performed at Eli Lilly using the Meso Scale Discovery (MSD) platform, and p-tau_231_ was quantified by ELISA [10]. For both the TRIAD and BioFINDER-2 cohorts, antibody-free mass spectrometry-based quantification of p-tau was performed using liquid chromatography-mass spectrometry (LC–MS) from a 300 ﻿µl sample as described previously [27]. Briefly, CSF samples of 250 µl were spiked with a 10 µl heavy isotope-labeled peptide standards (AQUA peptides, Thermo Scientific) mixture. The spike-in amount of each heavy peptide was modified to yield a light-to heavy peak area ratio of approximately 0.1 – 0.2 in CSF from subjects without AD. The peptide standards were diluted by mixing 10 pmol lyophilized aliquots with 20% acetonitrile. The final 1:10 dilution was performed in 50 mM ammonium bicarbonate to prevent acetonitrile interference during sample preparation. Protein precipitation was performed by adding perchloric acid (15 µl, 60% v/v) to the samples, which then were briefly vortexed and incubated on ice for 15 min. Under such circumstances, a majority of CSF proteins precipitate, though tau does not. The precipitated proteins were then pelleted by centrifugation at 30,000 × g for 10 min at 4 °C, and the supernatants were transferred to a 96-well filter microtitre plate (AcroPrep Advance, 350 µl, 0.45 µm, Supor membrane, Pall Corporation). A vacuum manifold was employed to pass samples through the filter plate and load them on a 96-well SPE plate (Oasis PRiME HLB 96-well µElution Plate, 3 mg Sorbent per Well, Waters). The SPE plate was washed in duplicate with 200 µl 5% methanol (v/v), and peptides were eluted into a microtitre plate with 200 µl 50% acetonitrile, 0.1% trifluoroacetic acid, and the eluates were lyophilized by vacuum centrifugation. Trypsin (Sequencing grade, Promega) was dissolved in the manufacturer diluent and diluted to 2.5 µg/ml in 50 mM ammonium bicarbonate. A 40 µl trypsin solution was added to the dry samples, which were then vortexed and incubated at 37 °C overnight. TFA (1 µl, 10% v/v) was added to the samples to quench additional proteolysis. The samples were then stored at -20 °C until LC–MS analysis. The tryptic peptides measured are described in the supplement. A parallel reaction monitoring (PRM) assay was used on a Hybrid Orbitrap mass spectrometer (Fusion Tribrid, Thermo Scientific). Single-point calibration was performed by adding internal heavy labeled peptides with the same sequence as the targeted peptides at a known concentration. Sample preparation took 2 days, with every sample requiring one hour to be analyzed in the Mass Spectrometer. These mass spectrometry measurements allow for the quantification of concentrations as low as in fmol/ml range, with an absolute precision of 0-2 ppm for quantified peptides. LC–MS data was analyzed using the Skyline v. 21 software package (MacCoss Lab, University of Washington, USA). Mass spectrometry-based measurements of p-tau residues in CSF were performed at the Clinical Neurochemistry Laboratory, University of Gothenburg by scientists blinded to clinical and biomarker information.

### PET imaging acquisition and processing

#### TRIAD

[^18^F]AZD4694 PET and [^18^F]MK6240 PET scans were obtained using a Siemens High Resolution Research Tomograph. [^18^F]AZD4694 PET images were obtained 40–70 min post- injection and reconstructed on a 4-dimensional volume with 3 frames (3 × 600 s), as previously described [28]. [^18^F]MK6240 PET images were acquired at 90–110 min post-injection and reconstructed on a 4-dimensional volume with 4 frames (4 × 300 s) [29]. MRI acquisition and processing has been described previously [30]. To minimize interference of meningeal spillover, [^18^F]MK6240 images were meninges-striped before they were blurred, as described previously [31]. [^18^F]AZD4694 standardized uptake value ratio (SUVR) maps were calculated using the whole cerebellum gray matter as the reference region and [^18^F]MK6240 SUVR maps were generated using the inferior cerebellar grey matter as a reference region. Spatial smoothing allowed the PET images to achieve an 8-mm full-width at half-maximum resolution. Amyloid-β SUVR from a neocortical region of interest (ROI) for each participant was estimated by averaging the SUVR from the precuneus, prefrontal, orbitofrontal, parietal, temporal, and cingulate cortices [28], with amyloid-β positivity defined as an [^18^F]AZD4694 above 1.55 [28]. Tau-PET SUVRs were calculate in regions comprising Braak stages I-IV as previously described [30].

#### BioFINDER-2

Details regarding PET image acquisition and processing in the BioFINDER-2 study have been documented previously [25]. Briefly, amyloid-PET and tau-PET scans were obtained using [^18^F]flutemetamol and [^18^F]RO948 radiotracers, respectively. Amyloid-PET binding was quantified using a standardized uptake value ratio (SUVR) with a neocortical meta-region of interest normalized to the cerebellar grey matter. Tau-PET binding was assessed within a meta-ROI covering temporal brain regions. The reference region used for tau-PET quantification was the inferior cerebellar cortex. Furthermore, tau-PET binding was also evaluated in regions comprising Braak stages I-IV as previously described [27].

### Statistical analyses

Statistical analyses were performed in R v4.1.1 and GraphPad Prism v9. CSF biomarkers (immunoassay and mass spectrometry) were compared between indicated groups by parametric *t*-test with FDR-correction for multiple testing. Effect sizes of group differences between amyloid-PET-positive and -negative individuals were determined using Cohen’s *d*. We also looked at the mean fold change between amyloid-PET-positive and -negative groups for all p-tau biomarkers. Bland–Altman analyses assessed the agreement between measurements from mass spectrometry and immunoassays. Area under the Receiver Operating Characteristic (ROC) curve values were calculated for all p-tau biomarkers. Nonparametric Spearman correlation coefficients were calculated for PET biomarker associations due to the non-normal distribution of the data. We selected PET biomarkers as the reference standard instead of clinical diagnosis in accordance to the biological definition of AD [2]. Comparison of correlation coefficients was performed using the cocor package in R and Zhou’s test was used to determine 95% CIs of differences [32]. Statistical differences in area under the ROC curves were tested with DeLong’s test using the pROC package in R [33].

## Results

### Participants

Demographic and clinical characteristics of all individuals in the study are reported in Tables 1 and 2. The mean age of all participants in the TRIAD cohort was 62.5 (SD = 17.3) and 57.8% were women. In the BioFINDER-2 cohort, the mean (SD) age of all participants was 69.1 (SD = 11.3) and 53.4% were women. The mean MMSE score of all participants was 27.5 (SD = 4.09) in the TRIAD cohort and 26.1 (SD = 4.56) in the BioFINDER-2 cohort. In the TRIAD cohort, the AD dementia group was slightly younger on average than the CU older adult (*p* < 0.001) and MCI (*p* < 0.001) groups, while in the BioFINDER-2 cohort, the CU older adult group was slightly younger than the MCI, AD, and non-AD neurodegenerative disease groups.

**Table 1:** Clinical and demographic characteristics of the TRIAD cohort

	**Young (*N* = 23)**	**CU (*N* = 74)**	**MCI (*N* = 36)**	**AD (*N* = 24)**	**OND (*N* = 16)**	**Overall (*N* = 173)**
**Sex**						
Female, n (%)	15 (65.2%)	45 (60.8%)	19 (52.8%)	11 (45.8%)	10 (62.5%)	100 (57.8%)
Male, n (%)	8 (34.8%)	29 (39.2%)	17 (47.2%)	13 (54.2%)	6 (37.5%)	73 (42.2%)
**Age, years**						
Mean (SD)	23.2 (2.58)	69.7 (8.17)	71.2 (7.47)	63.1 (6.69)	64.7 (8.33)	62.5 (17.3)
**Education, years**						
Mean (SD)	17.2 (2.40)	14.7 (3.53)	15.2 (3.11)	15.1 (3.34)	13.8 (3.62)	15.1 (3.38)
**MMSE**						
Mean (SD)	29.8 (0.52)	29.1 (1.04)	28.1 (1.74)	20.6 (5.56)	25.9 (5.58)	27.5 (4.09)
**APOE**ε**4 carriers**						
Noncarriers, n (%)	16 (69.6%)	50 (67.6%)	20 (55.6%)	7 (29.2%)	13 (81.3%)	106 (61.3%)
Carriers, n (%)	7 (30.4%)	24 (32.4%)	16 (44.4%)	17 (70.8%)	3 (18.8%)	67 (38.7%)
**Neocortical (**^**18**^**F)AZD4694 SUVR**						
Mean (SD)	1.20 (0.06)	1.50 (0.42)	2.03 (0.60)	2.31 (0.510)	1.23 (0.10)	1.66 (0.57)

**Table 2:** Clinical and demographic characteristics of the Bio-FINDER-2 cohort

	**CU (*N* = 149)**	**MCI (*N* = 47)**	**AD (*N* = 63)**	**OND (*N* = 95)**	**Overall (*N* = 354)**
**Sex**					
Female, n (%)	78 (52.3%)	20 (42.6%)	30 (47.6%)	61 (64.2%)	189 (53.4%)
Male, n (%)	71 (47.7%)	27 (57.4%)	33 (52.4%)	34 (35.8%)	165 (46.6%)
**Age, years**					
Mean (SD)	64.7 (13.5)	73.1 (7.52)	74.4 (6.70)	70.5 (9.36)	69.1 (11.5)
**Education, years**					
Mean (SD)	12.5 (3.48)	12.5 (3.73)	11.6 (4.06)	12.2 (3.46)	12.3 (3.61)
Missing	0 (0%)	1 (2.1%)	0 (0%)	0 (0%)	1 (0.3%)
**MMSE**					
Mean (SD)	29.0 (1.13)	26.6 (1.92)	19.6 (4.50)	25.5 (4.32)	26.1 (4.56)
**APOE**ε**4 carriers**					
Noncarriers, n (%)	79 (53.0%)	14 (29.8%)	20 (31.7%)	61 (64.2%)	174 (49.2%)
Carriers, n (%)	70 (47.0%)	32 (68.1%)	43 (68.3%)	34 (35.8%)	179 (50.6%)
Missing	0 (0%)	1 (2.1%)	0 (0%)	0 (0%)	1 (0.3%)
**Neocortical (**^**18**^**F)Flutametamol SUVR**					
Mean (SD)	1.01 (0.21)	1.50 (0.29)	1.66 (0.2)	0.958 (0.08)	1.11 (0.30)
Missing	11 (7.4%)	8 (17.0%)	59 (93.7%)	67 (70.5%)	145 (41.0%)

### Comparison of mass spectrometry and immunoassay p-tau differences according to amyloid-PET status

**Fig. 1:**
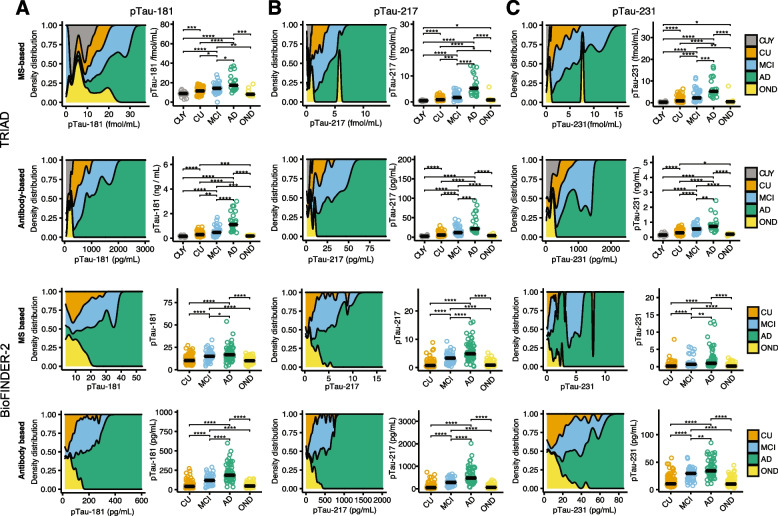
Distribution of immunoassay and mass spectrometry p-tau181, p-tau217 and p-tau231 concentrations by clinical diagnosis. Density plots represent the proportion of subjects (y-axis) at different concentrations of p-tau (x-axis) for mass spectrometry-based quantification of p-tau (top row & 3^rd^ row) and immunoassay-based quantification of p-tau (2.^nd^ row & bottom row). P-tau217 showed high specificity for AD with lower values for young adults and CU older adults. CUY = Cognitively unimpaired young adults; CU = Cognitively unimpaired older adults; MCI = Mild cognitive impairment; AD = Alzheimer’s disease; OND = Other neurodegenerative disease. *: *p* < 0.05; ** *p* < 0.01; *** *p* < 0.005; **** *p* < 0.001

Density and scatterplots displaying the distribution of p-tau_181_, p-tau_217_ and p-tau_231_ measured using immunoassay or mass spectrometry techniques are reported in Fig. 1. In the TRIAD cohort, mass spectrometry assessments of p-tau_181_ revealed that subjects with AD had relatively lower concentrations of p-tau_181_ as compared to when assessed using immunoassays. In contrast, mass spectrometry and immunoassay assessments of p-tau_217_ had quite similar distributions across diagnostic groups, except for one outlier with a non-AD neurodegenerative disease with high p-tau values assessed with mass spectrometry (but not with immunoassays) in the TRIAD cohort. The same subject also had high p-tau_231_ concentrations assessed with mass spectrometry, but not with immunoassays. A similar pattern of results was observed for the BioFINDER-2 cohort. A summary of fold changes, statistical comparisons, and effect sizes between amyloid-PET-positive and -negative groups for all p-tau biomarkers is reported in Tables 3 and 4.

**Table 3:** P-tau biomarker means, mean fold-change, statistical tests and effect sizes between amyloid-PET positive and negative groups in TRIAD

	**Aβ-**	**Aβ + **	**Log 2 Fold-change**	**Comparison** **t-value**	***p*-value**	**Effect Size**
Immunoassay p-tau_181_	6.14 (10.31)	22.5 (16.0)	1.55	15.97	< 0.0001	1.71
MS p-tau_181_	1.12 (0.51)	1.62 (0.55)	0.53	8.54	< 0.0001	0.98
Immunoassay p-tau_217_	291.52 (260.33)	855.85 (599.7)	1.87	20.17	< 0.0001	2.32
MS p-tau_217_	0.11 (0.19)	0.37 (0.27)	1.71	17.90	< 0.0001	2.21
Immunoassay p-tau_231_	281.82 (298.21)	651.27 (264.6)	1.21	17.06	< 0.0001	1.84
MS p-tau_231_	0.11 (0.24)	0.47 (0.32)	2.13	18.60	< 0.0001	1.97

P-tau biomarker means are reported in pg./ml for the immunoassays, and in fmol/ml for the MS assays. T-tests were carried out using log-transformed p-tau biomarker data. Effect sizes are reported as Cohen’s d

**Table 4:** P-tau biomarker means, mean fold-change, statistical tests and effect sizes between amyloid-PET positive and negative groups in BioFINDER-2

	**Aβ-**	**Aβ + **	**Log 2 Fold-change**	**Comparison** **t-value**	***p*-value**	**Effect Size**
Immunoassay p-tau_181_	42.23 (17.29)	118.44 (80.22)	1.49	11.68	< 0.001	1.48
MS p-tau_181_	10.36 (3.68)	14.81 (7.21)	0.52	5.83	< 0.001	0.84
Immunoassay p-tau_217_	53.76 (36.11)	273.05 (224.88)	2.34	13.98	< 0.001	1.55
MS p-tau_217_	0.78 (0.51)	2.98 (2.41)	1.93	11.87	< 0.001	1.42
Immunoassay p-tau_231_	10.99 (5.80)	26.28 (13.03)	1.26	12.33	< 0.001	1.65
MS p-tau_231_	0.20 (0.28)	1.20 (1.99)	2.60	9.73	< 0.001	0.80

P-tau biomarker means are reported in pg./ml for the immunoassays, and in fmol/ml for the MS assays. T-tests were carried out using log-transformed p-tau biomarker data. Effect sizes are reported as Cohen’s d

### Relationship between antibody-based and antibody-free p-tau concentrations

**Fig. 2:**
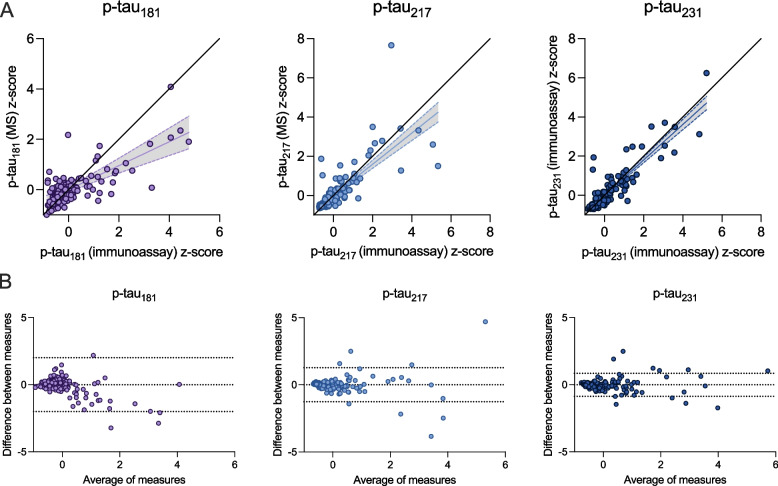
Correlations between immunoassay- and mass spectrometry-derived concentrations of p-tau in the TRIAD cohort. Top: Black lines of origin along the horizontal depict a theoretical linear relationship between variables without over- or under-estimation. The regression line and corresponding 95% confidence intervals below the origin indicates that mass spectrometry p-tau_181_ measurements underestimate p-tau concentrations from immunoassay measurements, which was not observed for p-tau_217_ or p-tau_231_. For p-tau_181_ in TRIAD (**A**), one data point (x = -0.45, y = 9.89) is not visually represented in the scatterplot in order to fit the plot to a comparable scale; this data point was nonetheless included in all analyses. Bottom: Bland–Altman analysis assessing bias between mass spectrometry and immunoassay measurements of p-tau. Dashed lines indicate limits of agreement, which correspond to standard deviation of the bias multiplied by 1.96. P-tau_231_ had the smallest bias, followed by p-tau_217_ and p-tau_181_ with the largest bias. Z-scores for each biomarker are represented to facilitate comparisons between measurements. MS = Mass spectrometry

**Fig. 3:**
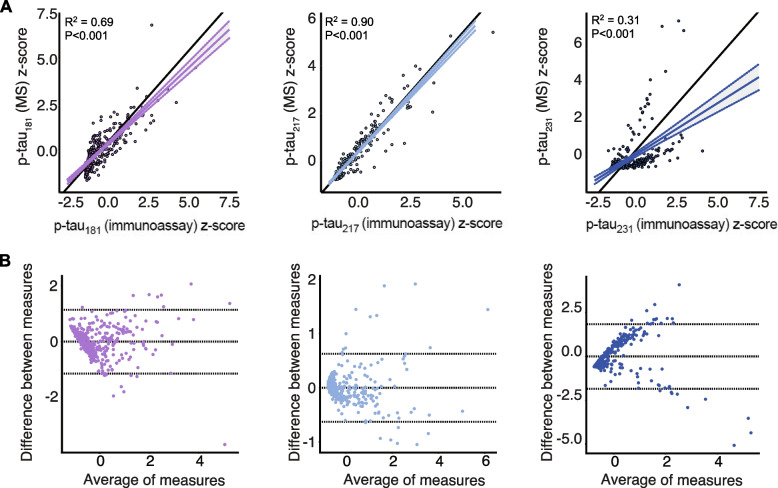
Correlations between immunoassay- and mass spectrometry-derived concentrations of p-tau in the BioFINDER-2 cohort. Top: Black lines of origin along the horizontal depict a theoretical linear relationship between variables without over- or under-estimation. The regression line and corresponding 95% confidence intervals below the origin indicates that mass spectrometry p-tau_181_ measurements underestimate p-tau concentrations from immunoassay measurements, which was not observed for p-tau_217_ or p-tau_231_. Bottom: Bland–Altman analysis assessing bias between mass spectrometry and immunoassay measurements of p-tau. Dashed lines indicate limits of agreement, which correspond to standard deviation of the bias multiplied by 1.96. P-tau_231_ had the largest bias, followed by p-tau_181_ and p-tau_217_ with the smallest bias. Z-scores for each biomarker are represented to facilitate comparisons between measurements. MS = Mass spectrometry

Scatterplots representing z-scored CSF p-tau biomarker concentrations from immunoassays and mass spectrometry are presented in Fig. 2A (TRIAD) and Fig. 3A (BioFINDER-2). For all three p-tau epitopes, a significant relationship was observed. In the TRIAD cohort, this linear relationship was strongest for p-tau_231_ (y = 0.90x – 0.005; *R*^2^ = 0.82, *p* < 0.0001) and p-tau_217_ (y = 0.79x + 0.004; *R*^2^ = 0.63, *p* < 0.0001) and weaker for p-tau_181_ (y = 0.47x + 0.008; *R*^2^ = 0.23, *p* < 0.0001), where mass spectrometry methods detected lower concentrations of p-tau_181_ compared with immunoassays. In the BioFINDER-2 cohort, p-tau_217_ had the strongest relationship when assessed with both antibody-free and antibody-based methods (y = 0.95x + 0.000; *R*^2^ = 0.90, *p* < 0.0001), while the relationships for p-tau_181_ (y = 0.83x + 0.000; *R*^2^ = 0.69, *p* < 0.0001) and p-tau_231_ (y = 0.56x + 0.000; *R*^2^ = 0.31, *p* < 0.0001) were weaker. Bland–Altman plots displaying the agreement between mass spectrometry and immunoassay p-tau biomarkers are presented in Fig. 2B (TRIAD) and Fig. 3B (BioFINDER-2). While substantial agreement was observed for all three p-tau analytes in the TRIAD cohort, p-tau_181_ had the largest standard deviation of bias (1.023), followed by p-tau_217_ (0.65) and p-tau_231_ (0.44). For all p-tau biomarkers, data points outside the upper and lower limits of agreement were more likely to be found at higher concentrations. Lower concentrations of p-tau_217_ and p-tau_231_ had values centered around 0 in Bland–Altman analyses, indicating very high agreement between measurements from mass spectrometry and immunoassays. The downward trend of datapoints in Fig. 2B for p-tau_181_ in TRIAD indicates that mass spectrometry systematically biased p-tau_181_ quantification, with larger magnitude of bias at higher concentrations. The Bland–Altman analyses in Fig. 3B for p-tau_231_ in BioFINDER suggests relatively lower agreement between antibody-based and antibody-free measurements.

### Associations of antibody-based and antibody-free p-tau concentrations with amyloid-PET and tau-PET

**Fig. 4:**
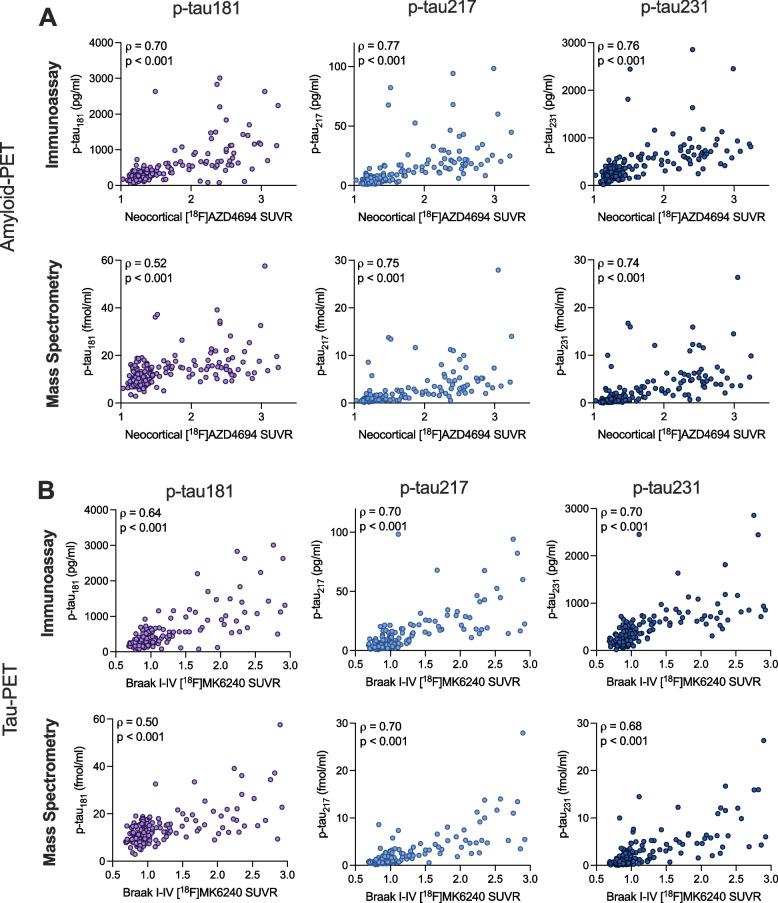
Relationship between immunoassay- and mass spectrometry-derived concentrations of p-tau with amyloid-PET and tau-PET in the TRIAD cohort. Scatterplots represent associations between p-tau181 (left), p-tau217 (middle) and p-tau231 (right) measured using both immunoassay and mass spectrometry with amyloid-PET (**A**), tau-PET (**B**). Correlation coefficients are presented as Spearman’s rho. P-tau_181_ measured with mass spectrometry had significantly lower correlations with both amyloid-PET and tau-PET than when measured with immunoassay (*p* < 0.001), whereas no differences were observed for p-tau_217_ or p-tau_231_. For p-tau_181_ measured with Mass Spectrometry, one data point (y = 119.4 fmol/ml) is not visually displayed to fit the axes on a comparable scale; this data point was nonetheless included in all analyses. A summary of all correlations, as well as comparisons between immunoassay and mass spectrometry is presented in Table 5

**Fig. 5:**
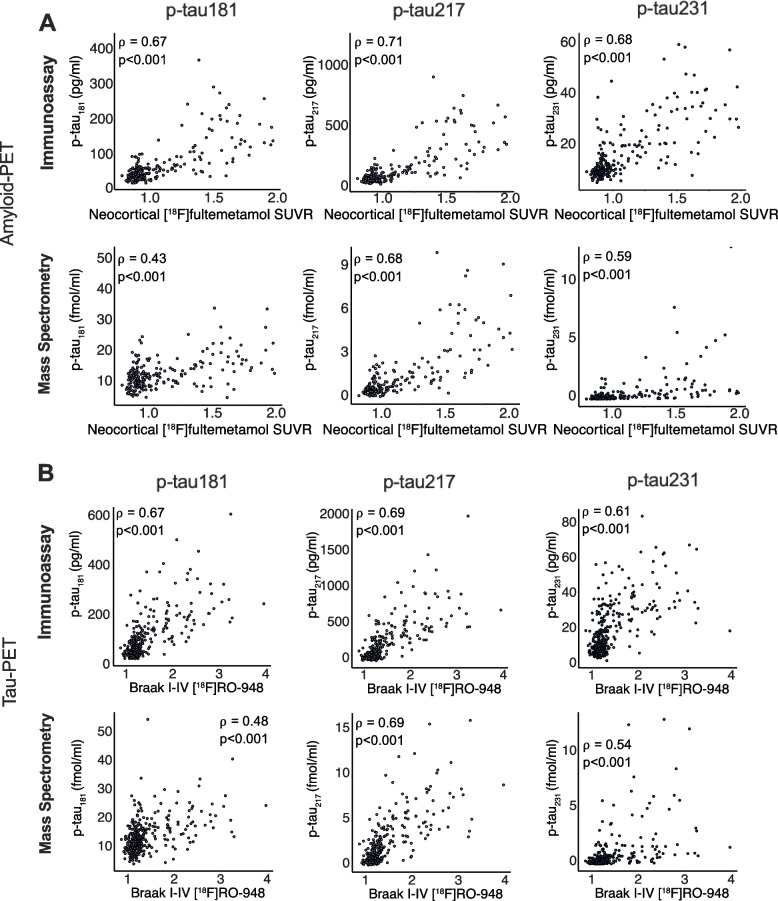
Relationship between immunoassay- and mass spectrometry-derived concentrations of p-tau with amyloid-PET and tau-PET in the BioFINDER-2 cohort. Scatterplots represent associations between p-tau181 (left), p-tau217 (middle) and p-tau231 (right) measured using both immunoassay and mass spectrometry with amyloid-PET (**A**), tau-PET (**B**). Correlation coefficients are presented as Spearman’s rho. P-tau_181_ measured with mass spectrometry had significantly lower correlations with both amyloid-PET and tau-PET than when measured with immunoassay (*p* < 0.001), whereas no differences were observed for p-tau_217_ or p-tau_231_. A summary of all correlations, as well as comparisons between immunoassay and mass spectrometry is presented in Table 6

Figure 4 displays the relationship between normalized p-tau concentrations measured with immunoassays vs. mass spectrometry with [^18^F]AZD4694 amyloid-PET and [^18^F]MK6240 tau-PET (TRIAD cohort). Figure 5 displays the relationship between CSF p-tau concentrations and [^18^F]Flutemetamol amyloid-PET and [^18^F]RO948 tau-PET (BioFINDER-2 cohort). Immunoassay assessments of p-tau_181_ displayed better fit with amyloid-PET than mass spectrometry-based assessments in both the TRIAD and BioFINDER-2 cohorts (*p* < 0.0001). Similarly, stronger associations with tau-PET were observed for immunoassay assessments of p-tau_181_ as compared with mass spectrometry (TRIAD *p* = 0.0008; BioFINDER-2 *p* < 0.0001). In contrast, no statistically significant differences were observed between immunoassay-based and mass spectrometry-based concentrations of p-tau_217_ for amyloid-PET (*p* = 0.42) tau-PET (*p* = 1.00) in the TRIAD cohort. Likewise, no statistically significant differences were observed between immunoassay-based and mass spectrometry-based concentrations of p-tau_231_ for amyloid-PET (*p* = 0.44) or tau-PET (*p* = 0.49) in the TRIAD cohort. In contrast, antibody-based assessments of p-tau_231_ had stronger relationships with both amyloid-PET (*p* < 0.001) and tau-PET (*p* < 0.001) in the BioFINDER-2 cohort than did antibody-free mass spectrometry measurements. A summary of statistical comparisons of correlations of immunoassay and mass spectrometry assessments of p-tau is provided in supplementary Table 1.

### Diagnostic performance antibody-based vs antibody-free p-tau concentrations

**Fig. 6:**
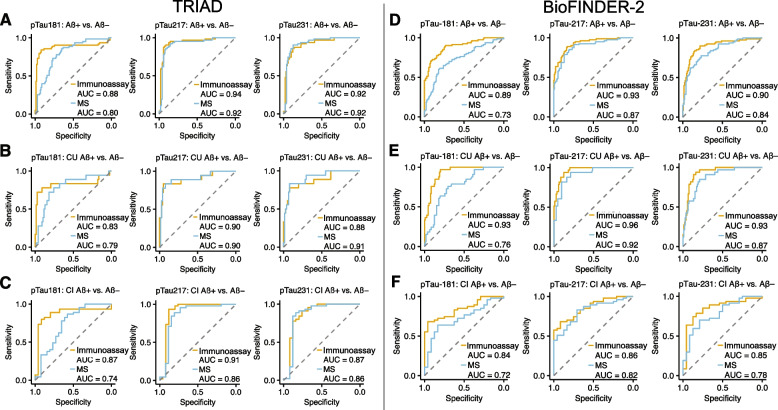
Discriminative accuracy of immunoassay- and mass spectrometry-derived p-tau concentrations for AD. ROC curves displaying discriminative accuracy of p-tau_181_ (left), p-tau_217_ (middle) and p-tau_231_ (right) measured using immunoassays (yellow lines) and mass spectrometry (blue lines) for amyloid-PET positivity. **A**, **D**: ROC curves for entire TRIAD and BioFINDER-2 samples. **B**, **E**: ROC curves for amyloid-PET positivity in CU individuals only. **C**, **F**: ROC curves for amyloid-PET positivity for CI individuals only. The summary of all statistical comparisons is reported in Tables 5 and 6. MS = Mass spectrometry;; CI = Cognitively impaired; CU = Cognitively unimpaired

Figure 6 displays ROC curves differentiating amyloid-PET-positive against -negative individuals using p-tau_181_, p-tau_217_ and p-tau_231_ concentrations measured with immunoassays and with mass spectrometry in the TRIAD and BioFINDER-2 cohorts. Mass spectrometry-based quantification of p-tau_181_ had significantly lower diagnostic accuracy for amyloid-PET positivity than p-tau_181_ measured from immunoassays in both the TRIAD and BioFINDER-2 cohorts. In contrast, no differences in diagnostic accuracy were observed for p-tau_217_ or for p-tau_231_ in the TRIAD cohort. In the BioFINDER-2 cohort, p-tau_217_ and p-tau_231_ antibody-free methods had marginally lower diagnostic accuracy for amyloid-PET positivity than p-tau_217_ (*p* = 0.023; 95% of difference: 1–7%) and p-tau_231_ (*p* = 0.052; 95% CI of difference 0–11%) measured with immunoassays. Similar patterns of results was observed when dividing the cohort into CU and CI subgroups. A summary of all area under the ROC curve values, corresponding 95% confidence intervals and statistical comparisons is presented in Tables 5 and 6.

**Table 5:** Area under the curve comparisons for plasma and CSF biomarkers for the identification of amyloid-PET positivity in the TRIAD cohort

	Immunoassay	MS	95% CI of difference	*p*-value
Amyloid-PET positivity in whole cohortsss				
p-tau_181_	88% (81–95%)	80% (73–87%)	1 – 17%	0.03
p-tau_217_	94% (90–98%)	92% (87–97%)	-3 – 3%	0.95
p-tau_231_	92% (87–96%)	92% (88–97%)	-4 – 3%	0.68
Amyloid-PET positivity in CU individuals				
p-tau_181_	83% (72–94%)	79% (69–88%)	-9 – 26%	0.33
p-tau_217_	90% (80–98%)	90% (79–98%)	-6 – 6%	0.99
p-tau_231_	88% (78–97%)	91% (81–99%)	-9 – 2%	0.25
Amyloid-PET positivity in CI individuals				
p-tau_181_	87% (77–96%)	74% (65–84%)	-1 – 20%	0.08
p-tau_217_	91% (81–98%)	86% (77–95%)	-1 – 11%	0.09
p-tau_231_	87% (78–97%)	86% (76–96%)	-5 – 8%	0.71

AUCs were compared using DeLong’s test. Values in parentheses represent 95% Confidence intervals

**Table 6:** Area under the curve comparisons for plasma and CSF biomarkers for the identification of amyloid-PET positivity in the BioFINDER-2 cohort

	Immunoassay	MS	95% CI of difference	*p*-value
Amyloid-PET positivity				
p-tau_181_	89% (84–94%)	73% (66–80%)	11 – 20%	< 0.000
p-tau_217_	93% (89–96%)	89% (84–94%)	1 – 7%	0.023
p-tau_231_	90% (85–94%)	84% (79–90%)	0 – 11%	0.052
Amyloid-PET positivity in CU individuals				
p-tau_181_	93% (89–97%)	76% (67–85%)	10 – 23%	< 0.000
p-tau_217_	96% (94–99%)	92% (87–97%)	1 – 8%	0.020
p-tau_231_	93% (89–97%)	87% (81–94%)	-1 – 12%	0.092
Amyloid-PET positivity in CI individuals				
p-tau_181_	84% (76–93%)	72% (60–84%)	4 – 20%	0.003
p-tau_217_	86% (78–94%)	82% (72–92%)	-2 – 10%	0.152
p-tau_231_	85% (75–95%)	78% (67–89%)	-5 – 18%	0.247

AUCs were compared using DeLong’s test. Values in parentheses represent 95% Confidence intervals

## Discussion

This study assessed the relationship between p-tau_181_, p-tau_217_ and p-tau_231_ quantified in CSF using immunoassays and with mass spectrometry. Diagnostic performance of antibody-free mass spectrometry p-tau_217_ and p-tau_231_ was comparable though marginally inferior to established immunoassay methods. However, p-tau_181_ quantified using mass spectrometry had inferior diagnostic performance and lower association with amyloid-PET and tau-PET than when measured using immunoassays. Taken together, our results suggest that diagnosis of AD using p-tau_217_ may be accomplished using either mass spectrometry or immunoassays, each having pros and cons. These particular mass spectrometry-based methods may also hold promise as candidate reference methods for absolute p-tau quantification in reference materials for assay standardization.

In contrast to p-tau_217_, p-tau_181_ and p-tau_231_ measured using mass spectrometry had inferior diagnostic performance and weaker associations with amyloid-PET and tau-PET. For p-tau_181_, this difference in performance is potentially attributable to a small phosphorylated endogenous tau peptide in CSF, which is identical to the peptide produced by trypsin cleavage that is measured in the mass spectrometry assay [34]. However, some of these p-tau forms are not detected using typical p-tau_181_ immunoassays which identify defined tau fragments, based on the antibody pair used, phosphorylated at the site of interest. These results suggest that phosphorylation of tau at threonine_181_ may have lower specificity for AD as compared to threonine_217_ or threonine_231_, when measured with mass spectrometry. In addition to differences in measurement techniques, there are slight differences in the analytes quantified from the immunoassay and mass spectrometry assays reported in this manuscript. The immunoassays used in this study target longer but defined tau peptides stretching from the N-terminus, using a partner antibody targeting the amino acids 6–18. In contrast, the mass spectrometry assays used in this study target shorter (9–15 amino acids) fragments of p-tau that have been cleaved with trypsin [27]. Specifically, in this study, the p-tau_181_ analyte is 15 amino acids long (175–190); the p-tau_217_ analyte is 9 amino acids long (212–221) and the p-tau_231_ analyte 15 amino acids long (225–240). Correspondingly, cleaving the tau peptide using trypsin may have resulted in a specific p-tau_181_ peptide not closely associated with AD that is not detected using immunoassays. Therefore, while mass spectrometry has the advantage of absolute quantification, it is important to consider that the intact tau protein itself is not being measured. Further investigation of different p-tau measurement techniques is warranted in light of findings that different tau biomarkers are non-interchangeable [35].

Despite the limitations of mass spectrometry described above, the antibody-free method described here addresses many limitations of immunoassay assessments of p-tau (or other analytes). Mass spectrometry-based methods may allow for quantifying multiple analytes in the same analytical run which stands to reduce sample analysis time as well as reduces the need to use multiple samples for each analyte [36]. Perhaps more importantly, inter-run variability can be reduced by lowering the number of freeze–thaw cycles [37]. Furthermore, inter-batch variability can be reduced by circumventing differences in antibody kits, which may enable more accurate quantification of longitudinal changes. Disadvantages of mass spectrometry include relatively low throughput and expensive instruments requiring personnel with a high level of expertise, batch-to-batch variation in the internal standard used to quantify the target peptide, gradually decreasing performance of the HPLC column, as well as unforeseen loss of diagnostic performance if tryptic peptides do not recapitulate the biomarker potential of larger forms of the protein.

The ability to measure multiple analytes concurrently using mass spectrometry may facilitate a more complete characterization of AD biomarker abnormality in a single subject. For example, given reported temporal ordering of p-tau biomarkers in CSF [12], understanding a patient’s abnormality at several tau phosphorylation sites has been hypothesized as a technique to stage AD severity [20, 38]. In fact, our study complements a recent report showing high performance of mass spectrometry-based quantification of p-tau_181_ and p-tau_217_ for discriminating between amyloid-PET positive and negative individuals [39]. Assessing multiple analytes from a single sample is an important advantage of biofluid assessments over imaging, which are highly specific to a single target.

Strengths of our study include a well-characterized research cohort with high-affinity PET imaging agents for amyloid-β plaques and tau neurofibrillary tangles, as well as out-of-sample replication in the BioFINDER-2 study. Our study also has several limitations. The first is that the TRIAD and BioFINDER-2 cohorts consist of self-selected individuals motivated to participate in a research study of aging and AD. The demographic makeup of this cohort is not representative of the populations at risk for dementia in North America or globally, therefore, replication of these results in more representative cohorts is a priority [40]. A second limitation is that this study focused on comparisons of immunoassays and mass spectrometry in CSF, replication of these results in plasma will be of critical importance given the very high performance of plasma p-tau_217_ for the diagnosis of AD [25, 41]. While studies have compared the diagnostic performance of plasma p-tau measured with mass spectrometry against more established immunoassay methods [42, 43], assessing their relationship with PET measurements of AD pathology will also be of substantial importance [44]. Furthermore, there are several practical concerns related to implementation of mass spectrometry or immunoassays that will determine the feasibility of either technique in clinical routine: cost of machine, cost of reagents, assay throughput, and analytical run time. In particular, for molecules with low abundance such as p-tau, mass spectrometry can be relatively slower. Correspondingly, future studies should compare advantages and disadvantages of these techniques before real-world implementation.

In conclusion, our study provides evidence that quantification of p-tau_217_ using immunoassays and antibody-free mass spectrometry have strong cross-sectional diagnostic performance and associations with amyloid-PET and tau-PET. Quantification of multiple analytes in a single run, which can be accomplished with mass spectrometry, may be a useful strategy to stage AD severity.

### Supplementary Information

**Additional file 1:Supplementary Table 1A. **Comparison of associations with Immunoassay and mass spectrometry assessments of p-tau with amyloid-PET and tau-PET in the TRIAD cohort. **Supplementary Table 1B. **Comparison of associations with Immunoassay and mass spectrometry assessments of p-tau with amyloid-PET and tau-PET in the BioFINDER-2 cohort.

**Additional file 2.**

## Data Availability

All requests for raw and analyzed data and materials will be promptly reviewed by McGill University to verify if the request is subject to any intellectual property or ﻿confidentiality obligations. Anonymized data will be shared upon request to the study’s senior author from a qualified academic investigator for sole the purpose of replicating the procedures and results presented in this article. Any data and materials that can be shared will be released via a material transfer agreement. Data are not publicly available due to information that could compromise the privacy of research participants. Related documents, including study protocol and informed consent forms, can similarly be made available upon request. Anonymized data from the BioFINDER-2 cohort study will be shared by request from a qualified academic investigator for the sole purpose of replicating procedures and results presented in the article and as long as data transfer agrees with EU legislation on the general data protection regulation and decisions by the Ethical Review Board of the cohorts, which should be regulated in a material transfer agreement.
