# Supplementary material for: Comparison of immunoassay- with mass spectrometry-derived p-tau quantification for the detection of Alzheimer’s disease pathology
Source: Mol Neurodegener. 2024 Jan 7;19:2. doi: 10.1186/s13024-023-00689-2 (PMC10773025; doi:10.1186/s13024-023-00689-2)
Supplement: Supplementary file 1 — Additional file 1:Supplementary Table 1A. Comparison of associations with Immunoassay and mass spectrometry assessments of p-tau with amyloid-PET and tau-PET in the TRIAD cohort. Supplementary Table 1B. Comparison of associations with Immunoassay and mass spectrometry assessments of p-tau with amyloid-PET and tau-PET in the BioFINDER-2 cohort. [file 13024_2023_689_MOESM1_ESM.docx]

**Supplementary Table 1A.** Comparison of associations with Immunoassay and mass spectrometry assessments of p-tau with amyloid-PET and tau-PET in the TRIAD cohort.

|  | Amyloid-PET correlations | | | | Tau-PET correlations | | | |
| --- | --- | --- | --- | --- | --- | --- | --- | --- |
|  | **Immunoassay** | **MS** | **95% CI of difference** | **p-value** | **Immunoassay** | **MS** | **95% CI of difference** | **p-value** |
| p-tau_181_ | 70% (61-77%) | 52% (39-62%) | 0.17 – 0.41 | <0.0001 | 64% (54-72%) | 50% (73-87%) | 0.09 – 0.33 | 0.0008 |
| p-tau_217_ | 77% (70-83%) | 75% (67-81%) | -0.07 – 0.16 | 0.42 | 70% (60-77%) | 70% (61-77%) | -0.10 – 0.10 | 1.00 |
| p-tau_231_ | 76% (68-82%) | 74% (66-80%) | -0.07 - 0.16 | 0.44 | 70% (62-77%) | 68% (59-75%) | -0.07 - 0.15 | 0.49 |

MS: Mass spectrometry. Values in parentheses represent 95% Confidence intervals.

**Supplementary Table 1B.** Comparison of associations with Immunoassay and mass spectrometry assessments of p-tau with amyloid-PET and tau-PET in the BioFINDER-2 cohort.

|  | Amyloid-PET correlations | | | | Tau-PET correlations | | | |
| --- | --- | --- | --- | --- | --- | --- | --- | --- |
|  | **Immunoassay** | **MS** | **95% CI of difference** | **p-value** | **Immunoassay** | **MS** | **95% CI of difference** | **p-value** |
| p-tau_181_ | 67% (58-75%) | 43% (29-54%) | 0.19 – 0.29 | <0.0001 | 67% (59-73%) | 48% (38-56%) | 0.14 – 0.24 | <0.0001 |
| p-tau_217_ | 71% (63-78%) | 68% (59-76%) | 0.01 – 0.05 | 0.02 | 69% (62-75%) | 69% (61-75%) | -0.03 – 0.03 | 0.99 |
| p-tau_231_ | 68% (58-76%) | 59% (48-67%) | 0.02 - 0.16 | 0.006 | 61% (53-69%) | 54% (46-61%) | -0.002 - 0.14 | 0.06 |

MS: Mass spectrometry. Values in parentheses represent 95% Confidence intervals.
