# Supplementary material for: Comparison of immunoassay- with mass spectrometry-derived p-tau quantification for the detection of Alzheimer’s disease pathology
Source: Mol Neurodegener. 2024 Jan 7;19:2. doi: 10.1186/s13024-023-00689-2 (PMC10773025; doi:10.1186/s13024-023-00689-2)
Supplement: Supplementary file 2 — Additional file 2. [file 13024_2023_689_MOESM2_ESM.docx]

**Comparison of immunoassay- with mass spectrometry-derived p-tau quantification for the detection of Alzheimer’s disease pathology**

**Supplementary material**

Tryptic peptides measured:

Thr-181: TPPAPK[pT]PPSSGEPPK 175-190

Thr-217: TPSLP[pT]PPTR 212-221

Thr-231: KVAVVR[pT]PPKSPSSAK 225-240
